# Overweight in childhood and consumer purchases in a Danish cohort

**DOI:** 10.1371/journal.pone.0297386

**Published:** 2024-03-12

**Authors:** Kathrine Kold Sørensen, Mikkel Porsborg Andersen, Frederik Trier Møller, Rikke Wiingreen, Marcella Broccia, Emil L. Fosbøl, Bochra Zareini, Thomas Alexander Gerds, Christian Torp-Pedersen

**Affiliations:** 1 Department of Cardiology, Nordsjællands Hospital, Hillerød, Denmark; 2 Division of Infectious Disease Preparedness, Statens Serum Institut, Copenhagen, Denmark; 3 Department of Pediatrics, Nordsjællands Hospital, Hillerød, Denmark; 4 Department of Obstetrics and Gynaecology, Aalborg University Hospital, Aalborg, Denmark; 5 Department of Paediatrics and Adolescent Medicine, Rigshospitalet, Copenhagen University Hospital, Copenhagen, Denmark; 6 Department of Cardiology, Rigshospitalet, Copenhagen, Denmark; 7 Department of Biostatistics, University of Copenhagen, Copenhagen, Denmark; Addis Ababa University School of Public Health, ETHIOPIA

## Abstract

**Background:**

Prevention and management of childhood overweight involves the entire family. We aimed to investigate purchase patterns in households with at least one member with overweight in childhood by describing expenditure on different food groups.

**Methods:**

This Danish register-based cohort study included households where at least one member donated receipts concerning consumers purchases in 2019–2021 and at least one member had their Body mass index (BMI) measured in childhood within ten years prior to first purchase. A probability index model was used to evaluate differences in proportion expenditure spent on specific food groups.

**Results:**

We identified 737 households that included a member who had a BMI measurement in childhood, 220 with overweight and 517 with underweight or normal weight (reference households). Adjusting for education, income, family type, and urbanization, households with a member who had a BMI classified as overweight in childhood had statistically significant higher probability of spending a larger proportion of expenditure on ready meals 56.29% (95% CI: 51.70;60.78) and sugary drinks 55.98% (95% CI: 51.63;60.23). Conversely, they had a statistically significant lower probability of spending a larger proportion expenditure on vegetables 38.44% (95% CI: 34.09;42.99), compared to the reference households.

**Conclusion:**

Households with a member with BMI classified as overweight in childhood spent more on unhealthy foods and less on vegetables, compared to the reference households. This study highlights the need for household/family-oriented nutrition education and intervention.

## Introduction

Childhood overweight and obesity is a global public health threat that affects more than one-third of children in Europe [1]. One major contributing factor is limited access to healthy, affordable foods and beverages, because of obesogenic food environments, including those found in supermarkets [2]. Prevention and management of childhood overweight and obesity at home is crucial for ensuring that children have a healthy start in life, as the challenges with overweight and obesity may require a focus on dietary patterns within the household [3]. Certain dietary patterns are associated with an increased risk of childhood overweight and obesity, including a high intake of saturated fat, free sugar, and salt, and a low intake of fruits, vegetables, and whole grains [2, 4]. Studies have demonstrated a correlation between low intake of fruits and vegetables and high intake of total fat in preschool-aged children [4]. Several reviews have shown that more energy-dense diets are associated with higher daily energy intake and subsequent weight gain, and that lowering intake of high-energy-density foods is an effective strategy for preventing overweight and obesity [5, 6]. Childhood overweight and obesity are associated with a range of morbidities and premature death, as well as an increased risk of sustained overweight and obesity in adulthood [7]. Further, childhood overweight and obesity are associated with a range of noncommunicable diseases such as diabetes [8], dyslipidemia, hypertension, liver disease, obstructive sleep apnea, polycystic ovary syndrome, and psychosocial issues [9, 10]. Studies have found that the food environment in the home can influence both food intake and weight in children [11] and that the availability of unhealthy foods in the home is associated with overweight and obesity among children in the household [12]. Consumer purchase data has been utilized to answer health-related questions dating back to as early as 1998 [13], however previous research has not been able to combine consumer purchase data with objectively measured anthropogenic information on a large scale. The purpose of this study was to characterize differences in household purchase patterns among households with at least one member who has had a Body Mass Index (BMI) classified as overweight or obese in childhood compared to reference households.

## Methods

### Study design and setting

This cohort study included all households who donated receipts and had at least one childhood BMI measurement recorded on one of the household members within 10 years of the first purchase. In Denmark, all citizens have equal access to healthcare services, including primary care and hospital access. A unique identification number, issued at birth or immigration, allows for individual-level linking of health and administrative databases. For this study, information was extracted from the following four registers through Statistics Denmark: 1) The Danish Civil Registration System [14] contains information on date of birth, gender, family type, municipality, and a unique household identification number. 2) The Danish National Child Health Register contains data on anthropometric measures taken since 2009 by trained health professionals during regular check-ups (from preschool to the end of primary school) for all children and adolescents in Denmark [15–17]. 3) The Income Statistics Register [18] holds information on income. 4) The Danish Education Register holds information on the highest attained education classified according to the International Standard Classification of Education (ISCED) [19]. Information on food purchases was retrieved from a digital receipt provider covering three of Denmark’s five largest retail chains. Households were identified by calendar year using the unique identification number. This allowed us to include all household members.

### Exposure

The Danish National Child Health Register includes information on BMI classified as thinness 3, thinness 2, thinness 1, normal, overweight, or obese based on sex- and age-specific curves established by the International Obesity Task Force [20]. Throughout, thinness 3, 2, and 1 are referred to together as underweight. Households with childhood were classified as having a member with childhood overweight or obesity if it included at least one member with a BMI measurement classified as overweight or obese at any point between ages 2–18 years. The reference households were all other households that had at least one member with a recorded BMI categorized as underweight or normal weight, throughout referenced to as the reference households.

### Outcome

Consumers donating receipts also shared their unique identification numbers, which allowed for linkage of the consumer purchase data with the registers at Statistics Denmark. The consumer purchase data was received on receipt level and included information on the product name, price, quantity, and date of purchase. The 487,340 unique product names were mapped to a food composition database [21] through regular expressions which allowed for categorization into 27 food groups. This mapping process and a list of foods and food groups have been described in more detail elsewhere [22, 23]. Only expenditures on edible goods were included in the analysis, and information on discounts was excluded due to uncertainty about which goods it applied to. The outcome of interest was the proportion of expenditures on certain food groups (salty snacks, confection, sugary drinks, ready meals, raw vegetables, and raw fruits) out of total expenditures in Danish Kroner, using supermarket receipts from January 1, 2019, to October 31, 2021. These food groups were chosen a priori from the 27 predefined categories.

### Covariates

Covariates were defined per household and calendar year prior to the first purchase. Information on date of birth, sex, family type, and municipality was collected from the Danish Civil Registration System, and the degree of urbanization was classified based on Eurostat’s Degree of Urbanization [24]. Family type was indicated as whether the household was a single household or not. Income was calculated as the five-year mean of the equivalized income within the household, accounting for redistribution within the family [18]. Education level was determined by the highest achieved education in the household, as recorded in the Danish Education Register [25] and classified into four groups based on the International Standard Classification of Education (ISCED): 0–2 (early childhood, primary education, and lower secondary education), 3 (general upper secondary education and vocational upper secondary education), 5–6 (short-cycle tertiary, medium-length tertiary, and bachelor’s-level education or equivalent), and 7–8 (second-cycle, master’s-level or equivalent, and PhD-level) [19]. Households with missing education information were assigned ISCED level 0–2.

### Statistical analyses

Baseline characteristics were summarized using median and quartiles for continuous variables, due to their non-normal distributions, and proportions for categorical variables. Consumer purchase data were summarized as total expenditure, total number of unique shopping trips, number of days between first and last shopping trip, and age of shopper at first purchase per household. A probabilistic index model [26] was used to estimate the probability that a random household with overweight or obesity spent a higher proportion on a specific food group, compared to a reference household, conditional on covariates. A probabilistic index of 50% defines no difference in the proportion expenditure distribution. The probability index model was adjusted for the following covariates: degree of urbanization, family type, highest attained education level in the family, and the five-year mean equivalized income to account for confounding. The analysis was repeated in subgroups of households where the shopper was the member with a BMI measurement in childhood and in subgroups of households where this was not the case, on the assumption that these may represent distinct types of households. The level of statistical significance was set at 5%. The statistical significance level was set at 5% and we controlled the family-wise error rate for multiple testing using the Bonferroni-Holm method [27]. Since records of childhood overweight and obesity could originate from up to ten years before the first purchase, misclassification is possible, and thus two sensitivity analyses were pre-specified and conducted where we redefined the criterion for the time of BMI measurement to seven and five years before the first purchase, respectively. Furthermore, we conducted a sensitivity analysis where households with underweight were excluded from the reference households. R Statistical software, version 4.2.1 was used for data management and analysis [28].

## Results

### Sample characteristics

Out of 11,882 households donating receipts, 93.8% were excluded due to a lack of BMI data. In total, 737 households were eligible for inclusion, 220 (29.9%) of the households were identified as including a member with childhood overweight or obesity and 517 (70.1%) with childhood underweight or normal weight. In 665 (90.2%) of the 737 households, the member who donated the receipts was the one who had their BMI measured as a child. The characteristics of the sample are shown in Table 1.

**Table 1:** Baseline characteristics of households. Baseline characteristics of the cohort, by whether the household included an individual with underweight or normal weight versus overweight or obesity.

Variable	Level	Underweight / Normal (n = 517)	Overweight/obesity (n = 220)	Total (n = 737)
Degree of urbanization, n (%)	Thinly populated	143 (27.7)	82 (37.3)	225 (30.5)
	Intermediate populated	144 (27.9)	69 (31.4)	213 (28.9)
	Densely populated	230 (44.5)	69 (31.4)	299 (40.6)
Family type, n (%)	Singles	282 (54.5)	92 (41.8)	374 (50.7)
	Couple	235 (45.5)	128 (58.2)	363 (49.3)
Educational level, n (%)	Basic education	94 (18.2)	52 (23.6)	146 (19.8)
	General upper secondary education	291 (56.3)	124 (56.4)	415 (56.3)
	Bachelor level education	91 (17.6)	31 (14.1)	122 (16.6)
	Masters or PhD	41 (7.9)	13 (5.9)	54 (7.3)
Income (Danish Krone*), n (%)	< = 124,000	169 (34.8)	56 (27.2)	225 (32.5)
	(124,000;248,000]	198 (40.7)	91 (44.2)	289 (41.8)
	(248,000;496,000]	111 (22.8)	56 (27.2)	167 (24.1)
	> 496,000	8 (1.6)	3 (1.5)	11 (1.6)
	missing	31	14	45
Total amount used in study period (Danish Krone)	median [IQR]	3202 [900, 8976]	3009 [828, 9401]	3108 [893, 9232]
N shopping trips	median [IQR]	43 [14, 97]	42 [12.5, 118.0]	43 [13, 102]
N days between first and last purchase	median [IQR]	349 [161, 634]	371.5 [144.0, 630.2]	354 [156, 631]
Age of shopper, years	< = 18	95 (18.4)	62 (28.2)	157 (21.3)
	(18,30]	399 (77.2)	147 (66.8)	546 (74.1)
	(30,45]	12 (2.3)	7 (3.2)	19 (2.6)
	> 45	11 (2.1)	4 (1.8)	15 (2.0)

Abbreviations: *IQR* interquartile range, *n* number, *SD* standard deviation

*100 Danish kroner approximate 13.44 Euro [44]

Most of the households with underweight or normal weight lived in densely populated areas (44.5%) whereas most of the households with overweight or obesity lived in thinly populated areas (37.3%). In both types of households, the most common family type was single, namely 54.5% of the households with underweight or normal weight and 41.8% of households with overweight or obesity. Both types of households had general upper secondary education (ISCED level 3) as the highest educational level in the households, approximately 56% for both. Similarly, in both types of households, most households had equivalized incomes ranging from 124,000 to 248,000 DKK, annually. Both households with and without childhood overweight or obesity had similar patterns of time between first and last purchase (approximately one year), amount spent in total (approximately 3,000 DKK), number of shopping trips (approximately 43), and distribution of age of the shopper.

### Marginal differences in proportion expenditure spent on specific food groups

**Fig 1:**
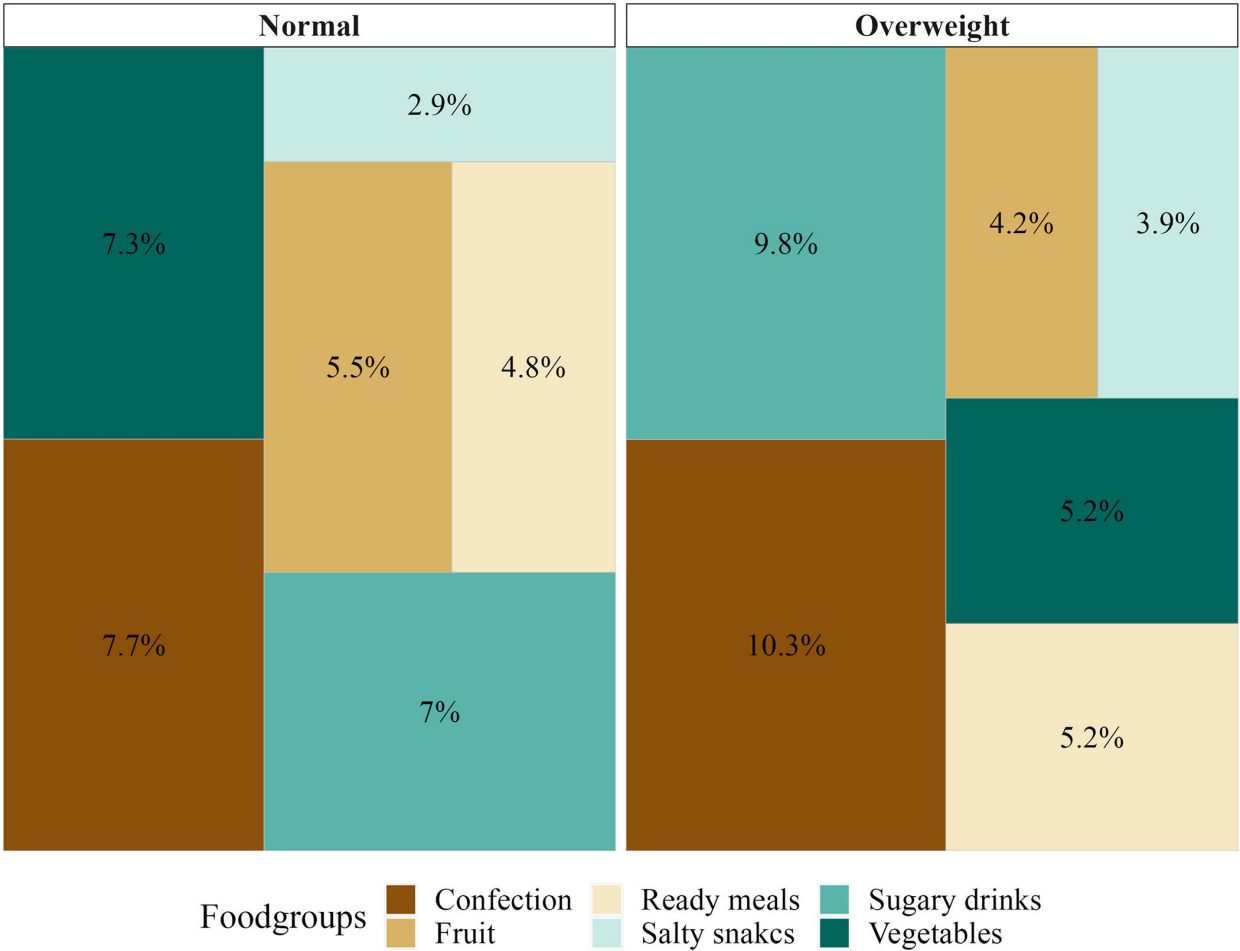
Childhood BMI and distribution of mean expenditure. Unadjusted distribution of mean expenditure spent on specific food groups, among households with and without a member who in childhood had a BMI classified as overweight or obese within the last ten years of first purchase.

Fig 1 visualizes the marginal proportion expenditure spent on the six food groups of interest out of the total expenditure spent on edible goods, for households with and without overweight or obesity, respectively.

On average, households with a member who in childhood had a BMI classified as overweight or obese spent a higher proportion of their expenditure on confectionery, ready meals, salty snacks, and sugary drinks, than households with underweight or normal weight. Conversely, households with a member who in childhood had a BMI classified as overweight or obese spent a lower proportion of their expenditure on raw fruits and vegetables than households with underweight or normal weight.

### Main analysis: Adjusted results of differences in proportion expenditure spent on specific food groups

**Fig 2:**
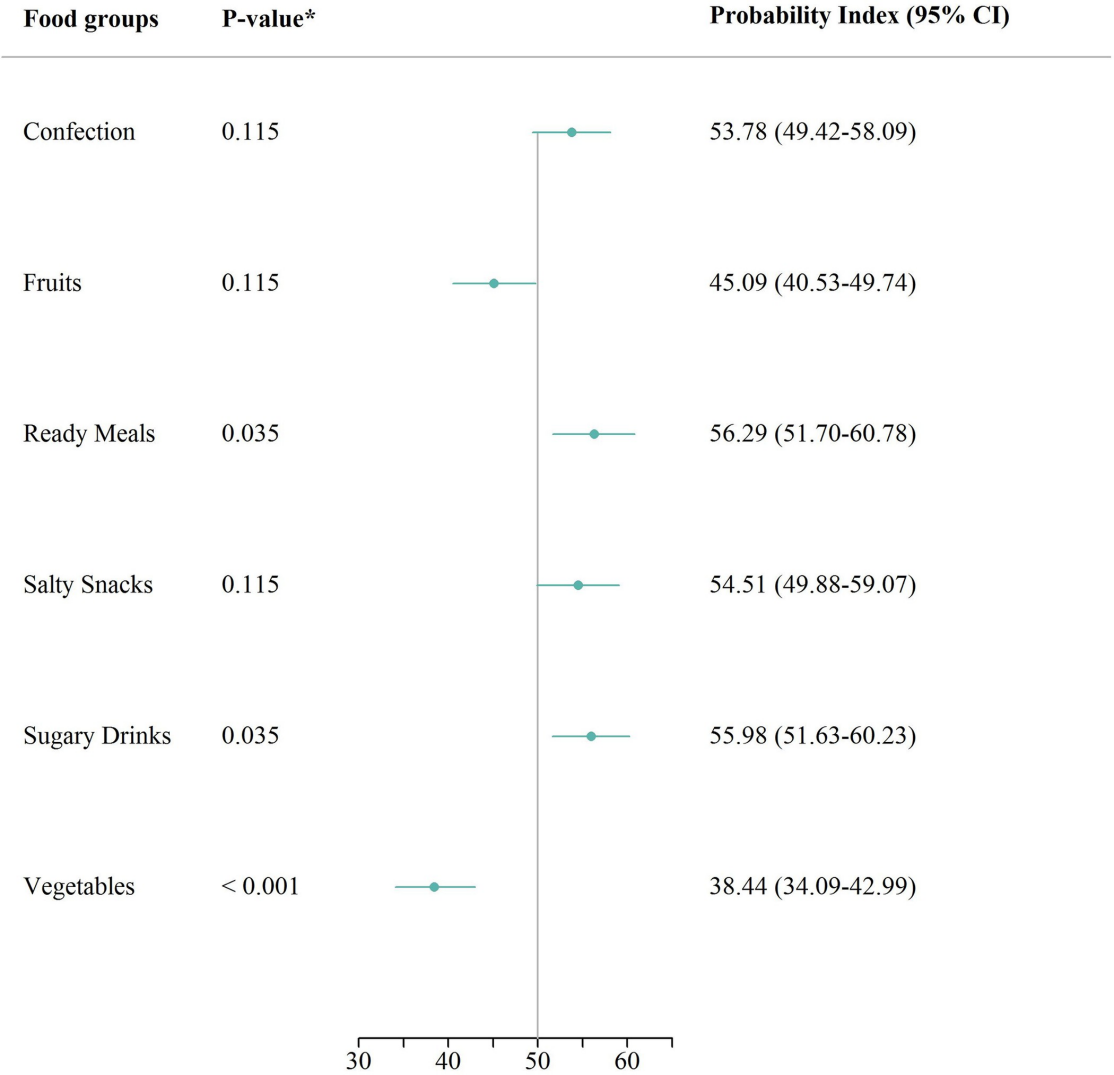
Conditional probability analysis: Childhood BMI and household purchases. Results of a conditional probability index model, estimating the probability that a household with a member who in childhood had a BMI classified as overweight or obese, purchased more of the specific good, compared to a reference household, conditional on highest attained education, five-year mean equivalized income, family type, and degree of urbanization, 50 indicating no difference. *Corrected using the Bonferroni-Holm method. Abbreviations: *CI* confidence interval.

The conditional results shown in Fig 2 indicate a statistically significant difference in the proportion expenditure spent between households with a member who as a child had a BMI categorized as overweight or obese and households without.

Accordingly, there was a statistically significantly higher probability that households with overweight or obesity had a higher proportion expenditure spent on Sugary drinks 55.98% (95% CI: 51.63;60.23) and ready meals 56.29% (95% CI: 51.70;60.78). Conversely, there was a statistically significant lower probability that households with overweight or obesity had a higher proportion of expenditure spent on vegetables 38.44% (95% CI: 34.09;42.99), compared to the reference households. Finally, although the results indicated no statistically significant difference in proportion expenditure spent on confection, fruits, and salty snacks, respectively, the estimates indicated that households with a member who as a child had a BMI categorized as overweight or obese had a higher probability of having a higher expenditure on confection 53.78% (95% CI: 49.42;58.09), Salty snacks 54.51% (95% CI: 49.88;59.07) and lower probability of a higher expenditure on fruits 45.09% (95% CI: 40.53;49.74), compared to the reference households.

### Secondary analysis: Adjusted results of differences in proportion expenditure spent on specific food groups in subgroups

When stratifying the analysis according to whether the shopper was a member with the childhood BMI measurement or not, similar effect estimates as the main analysis was found. Nevertheless, with one exception, the same tendency regarding the direction of the effect estimates was evident compared to the main analysis. The exact estimates are presented in Table 2.

**Table 2:** Probability of purchasing specific goods by households with a member in childhood classified as overweight or obese compared to a reference household.

	Households where shopper had BMI measurement: n = 665		Households where shopper was not the individual with BMI measurement: n = 73	
Food groups	P-value*	Probability index (95% CI)	P-value*	Probability index (95% CI)
Confection	0.153	54.07 (49.51;58.56)	1.000	53.9 (36.49;70.41)
Fruits	0.114	44.93 (40.23;49.72)	1.000	47.62 (23.37;73.05)
Ready meals	0.011	57.3 (52.55;61.92)	1.000	51.81 (30.03;72.93)
Salty snacks	0.153	54.33 (49.54;59.05)	1.000	49.67 (28.38;71.09)
Sugary drinks	0.010	57.24 (2.66;61.7)	0.838	62.78 (44.52;78)
Vegetables	< 0.001	39.71 (35.12;44.5)	0.002	6.99 (1.83;23.3)

*Corrected using the Bonferroni-Holm method

Abbreviations: *n* number, *CI* confidence interval, *BMI* body mass index

### Sensitivity analyses: Changing the number of years that the BMI measurement was allowed to be prior to first purchase

When changing the number of years that the BMI measurement was allowed to be prior to the first purchase to five and seven years, respectively, the number of included households diminished to 296 and 541, respectively, severely affecting the power of the estimates. However, with only one exception, the effect estimates were in the same direction and overall magnitude as the main analysis (S1 Table in S1 File). The results remained robust when excluding households with underweight from the reference households (S1 Fig in S1 File).

## Discussion

In this cohort study, households with a member with childhood overweight or obesity had an increased probability of spending a higher proportion expenditure on sugary drinks and ready meals, and a decreased probability of spending a higher proportion expenditure on vegetables, compared to the reference households. Stratification on households with versus without the shopper as the member with recorded childhood BMI did not change the estimates. Sensitivity analyses targeting possible misclassification did not change our interpretation of the results. Our finding of an increased probability of households with overweight spending more on sugary drinks and ready meals while conversely a decreased probability of spending more on vegetables is a concerning finding as sugary drinks and ready meals are often high in calories and low in nutrients [2, 5, 6], while vegetables are low in calories and high in nutrients. It is therefore recommended to increase the proportion of vegetables in the diet for challenges with overweight and obesity [2, 5, 6]. Our findings suggest that even years after challenges with overweight or obesity are present in the family, inexpedient tendencies in distribution of grocery expenditures persist. Beyond the hereditary component of suffering from overweight or obesity robustly demonstrated in a twin study [29], children’s’ acquired food preferences, and in turn weight, are also shaped by structures provided by the caretakers in the households [30]. Accordingly, studies have shown that children tend to consume readily available foods [31], with exposure increasing their preference for high energy foods [32, 33], emphasizing the impact of purchasing patterns on habits. Furthermore, children’s food-related knowledge, preferences, and consumption patterns are associated with their parents’ knowledge, preferences, and consumption patterns [32, 34, 35]. Specifically, it has been demonstrated that children’s intake of fruit and vegetables was positively related to parents’ intake of fruit and vegetables [36, 37]. Consequently, food-related structures in the household are essential in the development of food preferences and consumption habits, and the household food environment and in turn grocery purchases, represent a central sphere when it comes to improvement of children’s eating habits and prevention of obesity [38, 39] which support the findings in the present study, namely that household including a member who suffered from overweight or obesity in childhood have inexpedient grocery shopping patterns.

### Strengths and limitations

Inclusion in the study required a BMI measurement during childhood. Since BMI was only systematically registered from 2009 onward [15], the cohort was restricted to households including at least one member between 2 and 30 years of age. Taking the range of several years into account, it is unlikely that our results are affected by selection bias due to this restriction. BMI, as a measure, has limitations and its relevance as an indicator of overweight or obesity is a subject of ongoing debate. Namely, one study found that BMI underestimates the prevalence of overweight or obesity in adolescents [40]. However, in this study, approximately 30% of the households included a member who, as a child, had a BMI classified as overweight or obese, which aligns with expected numbers [41]. Likewise, our findings, indicating a higher prevalence of overweight or obesity in households situated in less urban areas, align with in other Western countries, where obesity rates are higher among rural children [42]. A central limitation is our lack of access to the entirety of the household grocery purchases, and it is important to note that household purchases may not fully capture individual intake, particularly when considering dining out. However, evidence exists that points to household purchases as a relevant proxy of individual intake [12, 43]. Further limitations include monetary expenditure used as a metric of comparison, not accounting for price dispersions among identical products regarding nutritional components. Mean proportion expenditure also does not account for the included households donating receipts over different periods, different lengths of time, and having different total expenditures over different frequencies of shopping trips. Regardless, households with and without overweight or obesity had similar medians of total amount used, number of shopping trips, and number of days between first and last purchase. Another limitation is that the mapping of information of the receipt to relevant food groups categorization is done based on a non-validated algorithm, albeit all matches were conducted using a food database maintained by an official and well-regarded institution [21] and were subsequently checked manually at least once. The results of this study should be considered in light of the fact that it only included households in which at least one member contributed with grocery receipts, which may constitute a selected group. Thus, the generalizability of the findings may be limited to such households, and caution should be exercised when extrapolating the results to other populations. One key strength is the use of objective data on household grocery purchases, which minimized bias that may be introduced through self-report methods such as food frequency questionnaires or 24-hour recall questionnaires. Objective estimation of household grocery purchase eliminates concerns related to recall bias or social desirability bias, which are common sources of systematic information bias in studies estimating household diet. Furthermore, we expect minimal or no systematic information bias in the measurement of BMI, as height and weight measurements were carried out by trained personnel in accordance with standardized and mandatory national preventive efforts, increasing the robustness of our findings.

### Implications

The current study adds to the evidence that inexpedient patterns of food purchases exist in households that have suffered, and perhaps still do, from challenges surrounding overweight or obesity. Malnutrition among children deserves attention and demands a family-oriented holistic approach including nutrition education and intervention. More ambitious applications of the findings may include integrating a continuous analysis of individual-level consumer purchase data into a consumer-oriented product that can provide feedback and guidance to the user.

### Conclusion

This study demonstrates the presence of unhealthy food purchase patterns in households with a member who had overweight or obesity in childhood. These households had a higher likelihood of spending a higher proportion of expenditure on unhealthy food groups and a lower likelihood of spending a higher proportion expenditure on healthy food groups, compared to the reference households. This study highlights the need for action toward family-oriented nutrition education and interventions, aimed particularly at increasing vegetables purchases and reducing the purchase of sugar-sweetened beverages and ready meals.

## Supporting information

S1 File (DOCX)
