# Supplementary material for: Overweight in childhood and consumer purchases in a Danish cohort
Source: PLoS One. 2024 Mar 12;19(3):e0297386. doi: 10.1371/journal.pone.0297386 (PMC10931458; doi:10.1371/journal.pone.0297386)
Supplement: S1 File — (DOCX) [file pone.0297386.s001.docx]

# Supplementary

**Table S1.** **Probability of purchasing specific goods by households with a member in childhood classified as overweight or obese compared to a reference household, where the BMI measurement was allowed to be <=5 years and <=7 years prior to first purchase, respectively**

|  | BMI measurement <=5 years prior to first purchase, n=296 | | BMI measurement <=7 years prior to first purchase, n=541 | |
| --- | --- | --- | --- | --- |
| Food groups | P-value* | Probability index  (95% CI) | P-value* | Probability index  (95% CI) |
| Confection | 1.000 | 48.04 (41.32;54.82) | 0.778 | 50.74 (45.62;55.84) |
| Fruits | 1.000 | 45.26 (38.10;52.61) | 0.753 | 47.45 (42.00;52.96) |
| Ready meals | 0.934 | 55.14 (48.04;62.04) | 0.233 | 55.14 (49.82;60.34) |
| Salty snacks | 1.000 | 50.31 (42.92;57.69) | 0.753 | 53.16 (47.76;58.48) |
| Sugary drinks | 1.000 | 51.87 (45.28;58.40) | 0.049 | 56.62 (51.60;61.51) |
| Vegetables | 1.000 | 45.69 (38.28;53.29) | <0.001 | 39.54 (34.38;44.93) |

*Corrected using the Bonferroni-Holm method

Abbreviations: *n* number, *CI* confidence interval, *BMI* body mass index

**Fig S1.** **Conditional Probability Analysis: Childhood BMI and Household Purchases excluding those with underweight.**

Results of a conditional probability index model, estimating the probability that a household with a member who in childhood had a BMI classified as overweight or obese, purchased more of the specific good, compared to a reference household, excluding those with underweight, conditional on highest attained education, five-year mean equivalized income, family type, and degree of urbanization, 50 indicating no difference. *Corrected using the Bonferroni-Holm method

Abbreviations: *CI* confidence interval
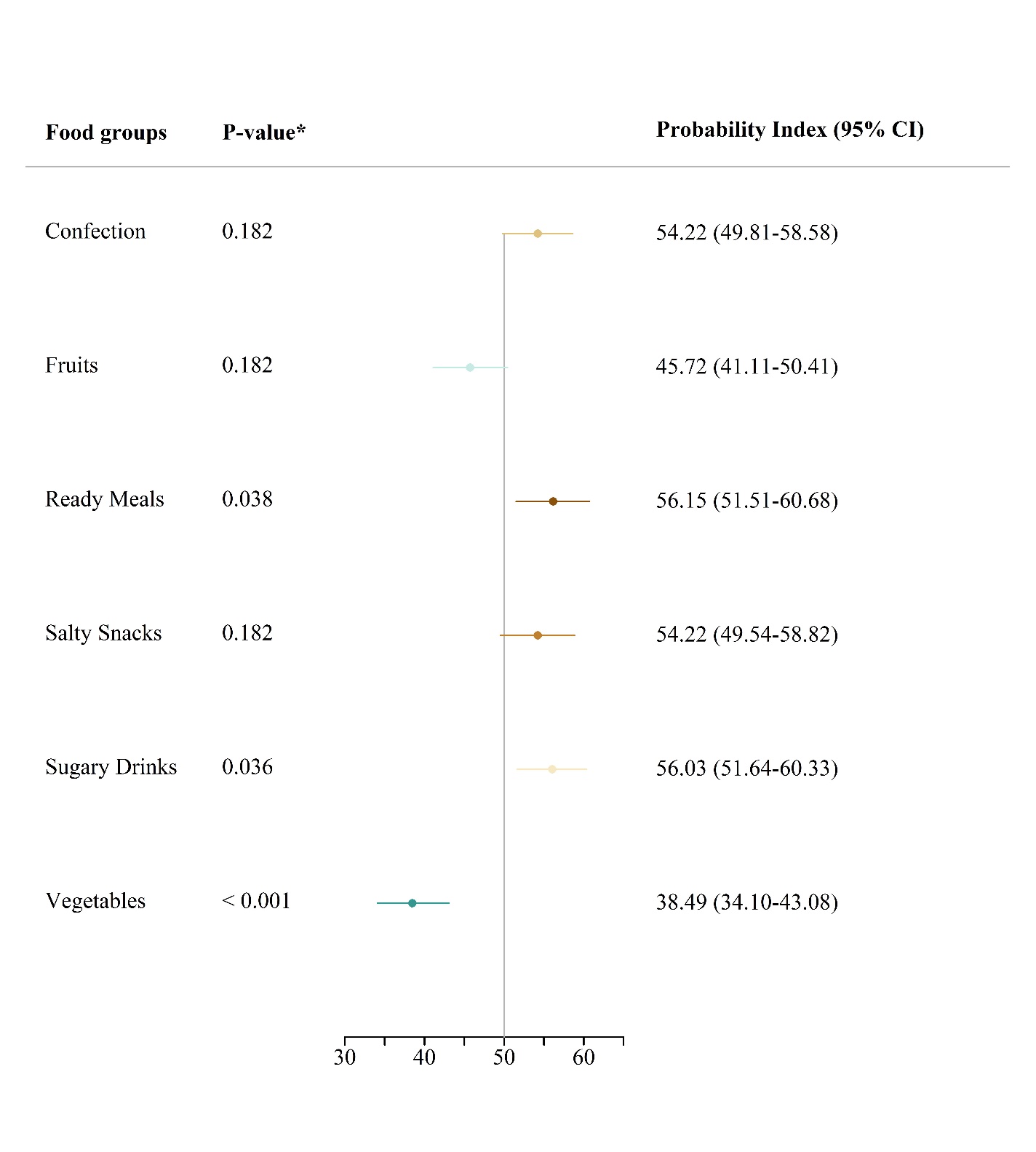


STROBE Statement—Checklist of items that should be included in reports of ***cohort studies***

|  | Item No | Recommendation | Page No |
| --- | --- | --- | --- |
| **Title and abstract** | 1 | (*a*) Indicate the study’s design with a commonly used term in the title or the abstract | 2 |
|  |  | (*b*) Provide in the abstract an informative and balanced summary of what was done and what was found |  |
| Introduction | | | |
| Background/rationale | 2 | Explain the scientific background and rationale for the investigation being reported | 3 |
| Objectives | 3 | State specific objectives, including any prespecified hypotheses | 4 |
| Methods | | | |
| Study design | 4 | Present key elements of study design early in the paper | 4 |
| Setting | 5 | Describe the setting, locations, and relevant dates, including periods of recruitment, exposure, follow-up, and data collection | 4 |
| Participants | 6 | (*a*) Give the eligibility criteria, and the sources and methods of selection of participants. Describe methods of follow-up | 4 |
|  |  | (*b*) For matched studies, give matching criteria and number of exposed and unexposed |  |
| Variables | 7 | Clearly define all outcomes, exposures, predictors, potential confounders, and effect modifiers. Give diagnostic criteria, if applicable | 5-6 |
| Data sources/ measurement | 8* | For each variable of interest, give sources of data and details of methods of assessment (measurement). Describe comparability of assessment methods if there is more than one group | 4-7 |
| Bias | 9 | Describe any efforts to address potential sources of bias | 4-7 |
| Study size | 10 | Explain how the study size was arrived at | 4 |
| Quantitative variables | 11 | Explain how quantitative variables were handled in the analyses. If applicable, describe which groupings were chosen and why | 5-6 |
| Statistical methods | 12 | (*a*) Describe all statistical methods, including those used to control for confounding |  |
|  |  | (*b*) Describe any methods used to examine subgroups and interactions | 6-7 |
|  |  | (*c*) Explain how missing data were addressed |  |
|  |  | (*d*) If applicable, explain how loss to follow-up was addressed |  |
|  |  | (*e*) Describe any sensitivity analyses |  |
| Results | | |  |
| Participants | 13* | (a) Report numbers of individuals at each stage of study—eg numbers potentially eligible, examined for eligibility, confirmed eligible, included in the study, completing follow-up, and analysed | 8 |
|  |  | (b) Give reasons for non-participation at each stage |  |
|  |  | (c) Consider use of a flow diagram |  |
| Descriptive data | 14* | (a) Give characteristics of study participants (eg demographic, clinical, social) and information on exposures and potential confounders | 9 |
|  |  | (b) Indicate number of participants with missing data for each variable of interest |  |
|  |  | (c) Summarise follow-up time (eg, average and total amount) |  |
| Outcome data | 15* | Report numbers of outcome events or summary measures over time | 10 |

| Main results | 16 | (*a*) Give unadjusted estimates and, if applicable, confounder-adjusted estimates and their precision (eg, 95% confidence interval). Make clear which confounders were adjusted for and why they were included | 10-11 |
| --- | --- | --- | --- |
|  |  | (*b*) Report category boundaries when continuous variables were categorized |  |
|  |  | (*c*) If relevant, consider translating estimates of relative risk into absolute risk for a meaningful time period |  |
| Other analyses | 17 | Report other analyses done—eg analyses of subgroups and interactions, and sensitivity analyses | 11-12 |
| Discussion | | | |
| Key results | 18 | Summarise key results with reference to study objectives | 12 |
| Limitations | 19 | Discuss limitations of the study, taking into account sources of potential bias or imprecision. Discuss both direction and magnitude of any potential bias | 14 |
| Interpretation | 20 | Give a cautious overall interpretation of results considering objectives, limitations, multiplicity of analyses, results from similar studies, and other relevant evidence | 15 |
| Generalisability | 21 | Discuss the generalisability (external validity) of the study results | 15 |
| Other information | | | |
| Funding | 22 | Give the source of funding and the role of the funders for the present study and, if applicable, for the original study on which the present article is based | 21 |

*Give information separately for exposed and unexposed groups.
